# Protecting Patient Privacy: A Suggested Framework for Deploying Extended Reality Technologies

**DOI:** 10.1177/29941520251400039

**Published:** 2025-12-02

**Authors:** Rick Beberman, Jesse Courtier, Darren Straker

**Affiliations:** 1 Sira Medical, San Francisco, California, USA.; 2 Legal Department, Stanford Health Care, Stanford, California, USA.

**Keywords:** HIPAA, PHI, patient privacy, patient data, compliance, security

## Abstract

As extended reality (XR) devices such as head-mounted displays, augmented reality glasses, computers, tablets, smartphones, and their underlying XR applications become widely deployed in HIPAA-regulated environments, clear guidelines are essential to prevent disclosure of protected health information that traditional health care IT security measures do not adequately address. Without proper safeguards, these technologies could expose sensitive patient data through various means, including visual displays, audio capture, data management, and processing, potentially undermining patient trust. This commentary is meant to provide recommendations in advance of more formal guidelines for protecting patient privacy when XR tools are used in clinical settings. Given the emerging nature of these technologies, addressing compliance proactively is crucial to avoid missteps that could undermine adoption and limit the technology’s potential in health care.

## Main Text

As extended reality (XR) devices such as head-mounted displays (HMDs), augmented reality glasses, computers, tablets, smartphones, and their underlying XR applications become widely deployed in HIPAA (Health Insurance Portability and Accountability Act)-regulated environments, clear guidelines are essential to prevent disclosure of protected health information (PHI) that traditional health care IT security measures do not adequately address. Without proper safeguards, these technologies could expose sensitive patient data through various means, including visual displays, audio capture, data management, and cloud-based processing, potentially undermining patient trust.

This commentary is meant to provide recommendations in advance of more formal guidelines for protecting patient privacy when XR tools are used in clinical settings. Given the emerging nature of these technologies, addressing compliance proactively is crucial to avoid missteps that could undermine future adoption and limit the technology’s potential in health care.

This article is organized into three areas of responsibility: hospitals/health systems, hardware manufacturers, and software developers. It is designed to address patient privacy within HIPAA-regulated environments, including surgical planning, patient education, diagnostic applications, pain management, rehabilitation, and therapy—in short, all applications where the end-users are either clinicians or patients, ultimately helping clinicians and administrators more effectively implement XR programs.

While some recommendations overlap to create multiple layers of security, certain items may be transferable between categories depending on specific organizational needs and capabilities.

## Hospital/Health System Responsibilities

As covered entities, hospitals and health systems have extensive HIPAA responsibilities.^1^ Key obligations with respect to PHI cover issues that can impact any data that can identify a patient.^2^ When it comes to XR, organizations must have security safeguards in place, provide proper staff training, ensure that third-party vendors comply with HIPAA, and report breaches promptly. This includes allowing patients access to their medical records and informing them how their information is being used.^3–6^

Given that XR devices function as mobile computing platforms, the following should be considered in conjunction with NIST (National Institute of Standards and Technology) SP 800-124.^7^ This standard addresses the management of security for mobile devices. Critical XR issues include:

### Network security and infrastructure

Health care organizations must implement a strong network security framework designed for XR devices and applications to ensure HIPAA compliance and efficient operations. Based on the technical specifications for XR devices and HIPAA compliance requirements, current mobile device management (MDM) policies provide a foundation for XR device deployment but require adaptation.^3^ These adaptations include:

- User configuration profiles enhanced for XR-specific settings, including brightness, contrast, field of view, volume limits, hand tracking, and protocols to reduce fatigue and motion sickness.
- Security update protocols that maintain device functionality and continuous compliance for a variety of items, including XR-specific HIPAA security assessments and audit trail maintenance.
- Content distribution systems that support secure delivery from tablets, computers, and cloud platforms to both clinicians and patients.
- Screen sharing and collaboration features with appropriate access controls.
- Low latency requirements for real-time interactions and high-bandwidth needs for immersive content delivery.
- Flexible authentication systems supporting both single sign-on and, where clinically appropriate, secure shared access.

### PC-Tethered device considerations

HMDs and other devices powered by personal computers present additional security challenges that require evaluation of the ecosystem among the devices and PCs. This includes securing the host computer, managing data flows among devices, and ensuring computer-based vulnerabilities do not compromise the devices.

### VPN and remote access challenges

Off-premises VPN access to XR content requires reviewing technical capabilities and compliance requirements. Organizations must verify that:

- VPN network architecture can support the bandwidth and latency requirements of XR devices and applications without compromising security.
- Device firmware and security patches are current across VPN-connected XR devices.
- Data processing and temporary storage during VPN sessions comply with HIPAA.

#### Storage and data management

Many of the considerations regarding whether XR data should be stored in the cloud or on-premises are similar to storing other data in HIPAA-regulated environments, such as accessibility, integration, cost, and data sovereignty. These measures will have to be extended to support XR. Choosing between on-site and cloud storage for XR data in HIPAA-regulated environments depends on the organization’s technical needs, compliance requirements, budget considerations, and tolerance for risk.

#### Physical security protocols

Health systems must establish secure storage procedures and asset tracking for all XR devices. This includes centralized registration of individual MAC (Media Access Control) addresses, remote attestation, and real-time compliance monitoring.^8^ Network access control lists should be maintained with the principles of least privilege to ensure access only for intended tasks. XR devices should remain on and be charged in a docking station that allows for the delivery of security updates. Automated deprovisioning mechanisms should be instituted in the event of a protocol breach. Audit trails must be maintained for all provisioning and deprovisioning systems. Signs of device compromise, such as physical tampering, must also be monitored. Regular protocol reviews should consider that implementation frameworks may differ depending on the asset and its use.

#### Data de-identification standards

Robust de-identification processes should be implemented wherever possible, encompassing all XR applications.

#### Zone-based usage policies

System boundaries should be designated within the hospital to distinguish areas where XR devices can operate with full functionality, limited functionality, or are prohibited. For example, the use of see-through or video pass-through HMDs and similar devices should be limited to enclosed areas, such as clinician meeting rooms, exam rooms, operating rooms, or private offices, to prevent the inadvertent recording of patients and others. If these HMDs are used outside these rooms, their functionality should be limited or disabled. Protocols for moving between zones should be established. The extent to which XR software applications create and record physical space-mapping data should also be considered and adjusted as needed.

#### Ongoing security assessment

Health care organizations must implement ongoing security evaluation programs designed for XR in HIPAA-regulated environments. Regular risk assessments and penetration testing should focus on XR-specific vulnerabilities, including ever-changing attack vectors created by XR technologies. Organizations must actively monitor cybersecurity alerts and guidance from CISA (Cybersecurity and Infrastructure Security Agency), the NIST Cybersecurity Framework, and other relevant authorities regarding XR security threats.

XR systems in health care must protect traditional PHI and new types of data generated by XR. These include biometric data, such as eye-tracking patterns, gesture recognition, physiological responses during XR interactions, pupil dilation, and balance metrics. Spatial tracking data, which show patient movement, room layouts, and environmental context, also need protection, as they can identify patients or reveal sensitive medical information.

The flow of XR data and the behaviors these systems capture require security that addresses both the volume and sensitivity of data generated during clinical and patient interactions.

Attention must focus on preventing cyberattacks, including data interception, user impersonation, and service disruption targeting critical XR devices and applications. Organizations should also assess weaknesses in an application’s design or code that permit vulnerabilities such as malicious XR applications and privilege escalation.

Other threats include virtual asset manipulation (unauthorized modification of medical visualization objects), reality distortion attacks (altering patient perception of treatment information), and presence spoofing (impersonation within therapeutic virtual environments).

The integration of machine learning (ML) components in XR applications increases these risks through adversarial ML attacks. Without mitigation strategies, these attacks can extract sensitive patient information from ML models, poison training data, or manipulate real-time decision-making systems. Traditional security measures often fail to address these ML-specific vulnerabilities.

Effective mitigation strategies may include layered security systems that combine data from multiple sources to confirm the accuracy of biometric and spatial data. Detection systems can establish baseline behavior patterns for specific clinical environments to identify deviations that indicate manipulation. Regular assessment protocols should evaluate cybersecurity controls, including standard security threat assessment against immersive manipulation and hardware exploitation. For additional details on preventing cyberattacks, see NIST 800-154, Guide to Data-Centric System Threat Modeling.^9^

A recent scoping review interestingly noted inferred patient data from virtual reality usage data to be the most significant threat to patient privacy from a cybersecurity perspective (Lake et al., 2024).^10^

#### End-to-end encryption

Under HIPAA, covered entities have primary responsibility for PHI encryption and ensuring compliance from both hardware and software vendors, including conducting thorough security assessments of these vendors. Health systems must implement HIPAA-compliant protocols for data transmission, storage, and access, utilizing current industry standards such as AES-256.^11^ They should also regularly conduct audits of encryption implementation and verify the encryption status. These requirements extend to VPN networks.

#### Comprehensive training programs

Develop training curricula covering the operation of devices, applications, HIPAA regulations, incident response, and third-party responsibilities. Training should address XR competency and privacy issues, making sure staff understand their responsibilities when using XR. Include both clinical and IT personnel in training programs.

#### Patient consent protocols

Clear procedures for obtaining patient consent should be established before using XR devices. Consent language should explain the technology and its impact on privacy. Depending on the use, topics specific to XR consent include the collection of data, such as patient anatomy and biometrics, analysis of physical and emotional responses, the use of data for research, and potential physical effects of using HMDs. As with typical patient consent, risks, benefits, and alternatives to XR should be discussed.

For any XR-related research, given the large amounts and types of data collected from XR devices and applications, extra care is needed to ensure de-identified data cannot be re-identified.

#### Breach notification

Establish clear guidelines and procedures for notifying affected patients and the Department of Health and Human Services in the event PHI is inadvertently disclosed.

#### Inclusion in the legal medical record

Establish whether recordings and models generated using XR devices are accessible to patients as a part of their legal medical record and how those representations will be stored in the electronic medical record and reproduced for release of information purposes. Establish whether other data, such as biometric data (eye tracking, gesture patterns, and physiological responses) and spatial tracking information, are also accessible to patients.

#### Business associate agreements

Assess organizational risk appetite beyond standard HIPAA Privacy and Security Rule provisions and incorporate in the business associate agreements, such as: prohibition on the use of PHI to advance an XR company’s commercial endeavors (e.g., only use your PHI to support improvements to your instance of the MR tool but not for further pecuniary gain with other customers) and prohibitions on the re-identification of PHI.

#### Data retention requirements

Consult with legal counsel on the appropriate retention period for XR-created outputs, as it may represent a new category of record. Furthermore, these retention periods may affect the availability of these XR-created outputs for any legal discovery proceedings and your organization’s ability to produce the record.

#### State-level privacy requirements

In addition to HIPAA’s requirements, consider the privacy protections afforded within your state, which may be more expansive. As an example, if the XR tool offers an AI component, disclosure of AI’s use in decision-making, or the avoidance of algorithmic discrimination may be required.

## Hardware Manufacturer Requirements

Manufacturers of XR devices would typically not have access to PHI while providing their hardware for use in a hospital but should provide support for protecting PHI. This often includes designing products that support HIPAA-compliant configurations, including security features, and providing best practices for health care implementations. Critical issues include:

### Robust access control systems

Implement multi-factor authentication to restrict device access to authorized users. Devices should automatically log off the current user when sensors detect someone else, unless configured for shared use. This prevents unauthorized access during user transitions.

### Automatic session management

Configure devices to automatically log out users after periods of inactivity, using timeouts based on clinical workflow and security policies.

### Comprehensive access logging

A comprehensive audit trail of device activity, including user identification, session duration, applications accessed, and PHI interactions, should be tracked to ensure transparency and accountability. These logs must be tamper-resistant and easily accessible for audit purposes. When deciding whether to store logs locally or in the cloud with a device manufacturer, consider HIPAA compliance, data sovereignty and control, ease of access, and infrastructure.

### Strong encryption implementation

As mentioned above, covered entities have a primary responsibility for encrypting PHI and ensuring vendor compliance with applicable standards. Nonetheless, XR device manufacturers must build encryption capabilities into their firmware that can be utilized by health systems and software developers who support HIPAA security standards, such as AES-256.^11^ While hardware manufacturers are not responsible for PHI, there have to be assurances that encryption occurs at the device level before any data leaves the XR device, including wireless transmissions and local storage.

### Camera controls

Default mechanisms should allow cameras to activate only within defined fields of view when XR devices are used in multi-patient environments. This limits capturing other individuals or sensitive information peripherally. Provide accessible controls for health care providers to disable cameras when moving through multi-patient areas. These capabilities should also apply to any computer or tablet used with an XR device.

### Audio management

Default mechanisms should include voice recognition and recording systems that can be spatially limited and easily disabled in multi-patient environments. Audio processing should use noise cancellation and directional microphone arrays to minimize capture of ambient conversations. These capabilities should also apply to any computer or tablet used with an XR device.

### Clear visual indicators

Use prominent indicators or visual cues to show when cameras or audio recordings are active. These indicators should be visible from multiple angles and hard to disable.

### Remote security management

Provide the ability to remotely lock devices or delete sensitive data in the event of theft, loss, or other security breaches. Include the ability to trigger security protocols and implement user notification systems for potential security incidents. Remote security management should be implemented using the appropriate NIST guidelines for MDM, such as SP 800-124.^7^

## Software Developer Considerations

Depending on the software application, developers may be considered “business associates,” especially if the developer’s application processes, stores, and transmits PHI.^12,13^ It’s important that developers working with MR applications in hospital settings address the following as part of delivery:

### Application-level access logging

Implement comprehensive logging in applications to track user interactions with PHI, such as data accessed, modifications made, and access duration. Logs should integrate with hospital audit systems and support compliance reporting requirements.

### Role-based access control

For clinician-facing devices, applications should be designed with systems that allow health care organizations to define user roles with specific levels of PHI access, such as to assigned patients or clinical responsibilities. Access controls should align with the principle of minimum necessary access and support dynamic permission changes based on clinical context. For patient-facing XR applications, PHI visibility should be limited to their own, access should be granted only to a treatment session for a specific program, and modifications to system and device settings need to be restricted.

### Intelligent PHI management

Develop systems to automatically identify and highlight PHI within applications, enabling real-time redaction or blurring of sensitive information. Implement edge computing solutions that process and extract only necessary data while discarding raw PHI.

### Strong encryption implementation

Ensure all PHI displayed, processed, or temporarily stored by applications is encrypted according to HIPAA standards such as AES-256.^11^ Encrypt data at multiple layers, including application-level encryption that stays secure even if system security is compromised. For more information on encryption, see the *End-to-End Encryption* section in Hospital/Health System Responsibilities above.

### AI development

If an application includes AI features that learn from clinical data, use privacy techniques and learning approaches to prevent PHI from entering training datasets or being sent to external systems for model improvement.

### Minimal data retention policies

Design applications to remove data as soon as practical, using retention periods based on organizational and regulatory requirements. Applications should ensure data cannot be recovered after removal. For patient-facing applications, transfer data from the device as soon as possible. For clinician-facing applications, retain data longer for active patient cases or when it may be used for clinical decision support.

### Gaze-based controls

Integrate eye-tracking technology to:

- Ensure applications process PHI only when the user’s attention is on themselves or the intended patient. The technology should obscure, blur, or disable sensitive functions when the user’s gaze shifts to family members, other patients, employees, vendors, or nonclinical areas.
- In conjunction with minimum data retention policies, eye movement biometrics that are recorded to enhance a viewing experience or perform a medical screening should be purged as soon as practical.

### Contextual awareness systems

Develop applications that understand clinical context and automatically adjust privacy settings based on location, procedure type, number of people present, or other environmental factors.

### Receive notice of changes to a health care entity’s privacy practices

Incorporate notice requirements into contractual arrangements to ensure that requirements for protecting patient privacy and the ability to comply with those protections remain aligned.

## Moving Forward

In this commentary, we provided considerations and recommendations for patient privacy considerations in medical XR. As these technologies evolve and hospital adoption increases, these considerations will also need to change. Further changes will also be influenced by real-world implementation experiences and any adjustments to current regulatory guidance.

## Authors’ Contributions

R.B.—Conceptualization, investigation, project administration, resources, validation, visualization, writing—original draft, writing—review and editing; J.C.—Investigation, resources, validation, visualization, writing—review and editing; D.S.—Investigation, resources, validation, visualization, writing—review and editing.

## Abbreviations

AES: Advanced Encryption Standard
BAA: Business Associate Agreement
CISA: Cybersecurity and Infrastructure Security Agency
EMR: Electronic medical record
HIPAA: Health Insurance Portability and Accountability Act
HMD: Head-mounted displays
MAC: Media access control
MDM: Mobile device management
ML: Machine learning
NACL: Network access control list
NIST: National Institute of Standards and Technology
PHI: Protected health information
PoLP: Principles of Least Privilege
SP: Special publication
VPN: Virtual Private Network
XR: Extended reality
